# Online orientation in early school grades: relationship with ADHD, boredom, concentration tendencies, and mothers’ parenting styles

**DOI:** 10.3389/fpsyg.2025.1592563

**Published:** 2025-07-07

**Authors:** Izumi Uehara, Yuji Ikegaya

**Affiliations:** ^1^Institute for Education and Human Development, Ochanomizu University, Tokyo, Japan; ^2^Department of Psychology, Ochanomizu University, Tokyo, Japan; ^3^Graduate School of Pharmaceutical Sciences, The University of Tokyo, Tokyo, Japan

**Keywords:** online orientation, school children, ADHD tendency, boredom tendency, concentration tendency, mothers, maternal parenting style

## Abstract

This study investigated factors associated with online orientation and preferences in lower-grade schoolchildren, focusing on attention-deficit/hyperactivity disorder (ADHD), boredom, and concentration tendencies in both children and mothers, as well as maternal parenting styles. Data were collected from 341 mothers (172 of boys, 169 of girls), who completed rating scales on these factors and reported their children’s preferred activities and those they could concentrate on for extended periods. Based on maternal responses, children were categorized into “online-concentrated” (*n* = 191) vs. “non-online-concentrated” (*n* = 150) and “online-play” (*n* = 95) vs. “non-online-play” (*n* = 246) groups. ADHD and boredom tendencies in children were strongly associated with an online orientation, while concentration tendencies were linked to a non-online orientation. Maternal boredom tendencies also appeared to influence children’s online orientation. Furthermore, higher maternal control was associated with increased engagement in non-online activities. These findings imply that parents should tailor their approach to managing children’s online activities based on their children’s individual traits (e.g., boredom and ADHD tendencies) while also considering their own behavioral tendencies, such as boredom.

## 1 Introduction

Internet addiction (IA), characterized by compulsive internet use that detrimentally affects daily life and mental health, has come to be widely recognized as a serious societal issue. Existing studies have implicated several factors in the development and maintenance of IA, including neurodevelopmental disorders such as autism spectrum disorder (ASD) and attention-deficit/hyperactivity disorder (ADHD), parenting styles, and individual characteristics such as boredom proneness.

ASD and ADHD have both been associated with IA. Adolescents with ASD—particularly those exhibiting ADHD symptoms—appear to be at elevated risk for IA. In young adults, ADHD symptoms have been found to mediate the relationship between ASD and IA (Kawabe et al., 2019; Lyvers et al., 2024). Furthermore, ADHD symptoms have been identified as a significant risk factor for IA among both college and middle school students (Sahimi and Abd Latif, 2023; Wang et al., 2024; Weinstein et al., 2015).

Parenting styles (PS) may influence children’s risk of developing IA. Permissive PS has been linked to IA in preadolescents (Lo et al., 2020), while authoritarian PS increases IA risk among high school students (Bilge et al., 2022). Low family support and parental care have been found to indirectly influence IA by affecting children’s mental health (Chen et al., 2015; Trumello et al., 2021). Low parental supervision and lack of strictness have also been identified as predictors of IA in adolescents (Karaer and Akdemir, 2019). However, the way PS influences IA may vary depending on individual characteristics. Among adolescents and young adults, the effects of authoritarian PS on IA are moderated by high social intelligence (Ugwu et al., 2023), and life satisfaction influences the relationship between PS and IA (Liu et al., 2024).

Additionally, boredom proneness, a characteristic associated with ADHD symptoms, may contribute to IA. Boredom and understimulation have been shown to increase the risk of IA among adolescents and college students (Biolcati et al., 2018; Chou et al., 2018; Wang, 2019).

Although these previous findings suggest that IA is influenced by a complex interplay of individual characteristics (e.g., boredom proneness, ASD and ADHD symptoms) and parenting styles, parental traits—and their interaction with parenting styles—may also shape a child’s risk of developing IA. These effects may partly operate through child characteristics such as ADHD symptoms. Furthermore, there is evidence that maternal parenting styles may partially contribute to the development and manifestation of ADHD symptoms in children (Yoshimasu, 2020), highlighting the importance of considering these influences within a comprehensive developmental framework. These factors should be further examined to clarify the mechanisms underlying the development of IA and ADHD. Moreover, the potential reciprocal effects of IA on these variables may also need to be explored. The online environment may contribute to the manifestation of ADHD and ASD symptoms by masking core difficulties and fulfilling an increased need for external stimulation.

While most studies have focused on adolescents and young adults, IA may become increasingly prevalent among younger children as access to digital devices expands. Accordingly, this study exploratorily examined the relationship between children’s online orientation and preferences and several factors, including both children’s and mothers’ ADHD tendencies, boredom, and concentration tendencies (conceptualized as the cognitive opposite of boredom), and maternal PS. To operationalize online orientation and preferences as variables, children were categorized into “online” and “non-online” groups based on (1) whether the activity they could concentrate on most was online or offline, and (2) whether their favorite type of play was online or offline. We hypothesized that if a subset of children showed a strong inclination toward online activities despite preferring offline play, this discrepancy could highlight the influence of online platforms. Such findings would underscore the importance of implementing platform-level measures to help prevent IA in early childhood.

In Japan, mothers overwhelmingly assume the primary role in childcare, including play-related activities, making them the most reliable source of information regarding children’s daily behaviors (Sato, 2015; Tsuru and Kume, 2018). To ensure consistency in the source of parental data across variables, this study limited parental measures to maternal reports.

## 2 Methods

### 2.1 Participants

An email containing an overview of the study, honorarium details, and an invitation to participate was distributed through a commercial survey company to all registered parents of children in first through third grade. Questionnaires and consent forms were mailed to those who agreed to participate. Data were collected from parents who returned both the completed survey and signed informed consent forms by the specified deadline. A total of 341 mothers participated in the study, including 172 mothers of boys and 169 mothers of girls. The sample comprised middle-class mothers aged 30 to 50 years. In Japan, approximately 90% of respondents have long identified as middle class (Wang, 2020). Thus, using a survey company whose registrants are predominantly middle class aligns with national demographic trends.

Data with missing or incomplete values were excluded from the analyses. Although some participants did not report their age and six mothers provided slightly older-than-expected ages for their children, their data were included, as eligibility had been confirmed prior to participation. The mean ages of mothers of boys (*n* = 167) and girls (*n* = 166) were 40.61 ± 4.63 years and 40.76 ± 4.76 years, respectively, with no significant difference between the groups (t[331] = −0.290, *p* = 0.772). Similarly, the mean ages of boys (*n* = 169) and girls (*n* = 169) as reported by their mothers were 8.23 ± 0.94 years and 8.27 ± 0.95 years, respectively, with no statistically significant difference (t[336] = −0.387, *p* = 0.706). All participants were native Japanese speakers.

### 2.2 Materials and procedures

Participants completed several standardized scales and responded to two open-ended questions. The standardized instruments included:

1. The Japanese version of the Boredom Proneness Scale (BPS), consisting of 28 items rated on a seven-point scale (Farmer and Sundberg, 1986; Uehara et al., 2024).
2. The Japanese version of the Adult ADHD Self-Report Scale v.1.1, comprising 18 items rated on a five-point scale (Kessler et al., 2005).
3. The Japanese Parenting Style Scale, including 16 items rated on a four-point scale (Nakamichi and Nakazawa, 2003).
4. The Japanese version of the ADHD Rating Scale-IV for children, with 18 items rated on a four-point scale (DuPaul et al., 2008).
5. A single-item measure assessing the mother’s and child’s daily boredom tendency, rated on a seven-point scale.
6. A Single-item measure assessing the mother’s and child’s daily concentration tendency, rated on a seven-point scale.

Additionally, participants answered two open-ended questions:

1. What activities can your child concentrate on for a long time?
2. What are your child’s favorite play activities?

Data from 301 mothers concerning ADHD tendencies, boredom tendencies, and maternal parenting were also used in a separate study. However, that study did not include responses to the open-ended questions or the items assessing concentration tendencies. Instead, it incorporated additional variables, some of which were aggregated across parents, for distinct research purposes (Zhang, Ikegaya, and Uehara, submitted). The validity and reliability of the Japanese BPS were confirmed prior to analyses using BPS-related data from this study, along with responses from a separate sample of participants who completed the Japanese BPS twice (Uehara et al., 2024).

All procedures in this study were approved by the Humanities and Social Sciences Research Ethics Committee of Ochanomizu University (ethics approval number: 2021-152). Written informed consent was obtained from all participants prior to data collection.

### 2.3 Data coding of open-ended questions

Responses to the open-ended questions were categorized based on the primary activity type reported by mothers.

For activities in which children were perceived to concentrate for extended periods, those whose mothers first identified online content (e.g., digital games, smartphones, and YouTube) were assigned to the *online-concentration group* (*n* = 191, mean age = 8.34 ± 1.01 years). Children whose mothers first identified non-online content(e.g., outdoor play, reading, and sports) were categorized as the *non-online-concentration group* (*n* = 150, mean age = 8.14 ± 0.85 years).

Regarding children’s favorite play activities, those whose mothers reported online content first were classified into the *online-play group* (*n* = 95, mean age = 8.47 ± 1.07 years), whereas those whose mothers identified non-online content first were categorized as the *non-online-play group* (*n* = 246, mean age = 8.17 ± 0.88 years).

### 2.4 Statistical analyses and utilization of large language models (LLMs)

Statistical analyses were conducted using IBM SPSS Statistics, version 29. Language refinement of the manuscript was supported by ChatGPT, which was used to enhance grammatical accuracy and improve clarity of expression in English.

## 3 Results

### 3.1 Reliability of the scales

Total scores on the BPS, the Adult ADHD Self-Report Scale, and the ADHD Rating Scale for children were used in the analyses. The Cronbach’s alpha coefficients for these scales were 0.77, 0.89, and 0.94, respectively, indicating adequate to excellent internal consistency. Since the PS scale comprises two factors—responsiveness and control—we confirmed this factor structure within our dataset. The Cronbach’s alpha coefficients for these subscales were 0.75 for responsiveness and 0.61 for control, reflecting adequate and acceptable internal consistency, respectively (Nunnally and Bernstein, 1994). For these two factors, mean scores were used in the analyses (Nakamichi and Nakazawa, 2003).

### 3.2 Descriptive statistics

Tables 1 and 2 report the descriptive statistics for all variables, grouped by gender. Table 1 compares the online-concentrated and non-online-concentrated groups, while Table 2 compares the online-play and non-online-play groups. Although the online-play group was smaller in size, a higher proportion of boys was found in both online groups [Table 1: χ^2^(1) = 5.41, *p* = 0.020, adjusted residuals for all cells *p* < 0.05; Table 2: χ^2^(1) = 17.03, *p* < 0.001, adjusted residuals for all cells *p* < 0.01].

**Table 1:** Descriptive statistics for online-concentrated and non-online-concentrated groups.

Dependent variable	Group				*F(*χ^2^*)* and *p* values		
	Online-concentrated group		Non-online-concentrated group		Main group effect	Main gender effect	Interaction
	Boys	Girls	Boys	Girls			
Gender (number)	*n* = 107	*n* = 84	*n* = 65	*n* = 85	χ^2^ = 5.41, ***p*** = 0.020* (Distribution of the numbers of boys and girls)		
**C_Boredom proneness**	4.36 (± 1.78)	4.25 (± 1.66)	3.35 (± 1.67)	3.47 (± 1.72)	***F* = 22.52, ***p*** < 0.001** **	*F* = 0.000, *p* = 0.995	*F* = 0.376, *p* = 0.540
**C_Concentration tendency**	5.45 (± 1.34)	5.21 (± 1.19)	6.02 (± 1.18)	5.85 (± 1.23)	***F* = 19.10, ***p*** < 0.001** **	*F* = 2.152, *p* = 0.143	*F* = 0.058, *p* = 0.810
**C_ADHD tendency**	13.05 (± 11.26)	8.88 (± 8.00)	8.82 (± 9.74)	6.27 (± 6.24)	***F* = 11.60, ***p*** < 0.001** **	***F* = 11.16, ***p*** = 0.001** **	*F* = 0.651, *p* = 0.420
**M_BPS**	102.31 (± 15.28)	100.38 (± 13.05)	95.71 (± 16.41)	97.89 (± 16.36)	***F* = 7.319, ***p*** = 0.007** **	*F* = 0.006, *p* = 0.939	*F* = 1.500, *p* = 0.222
**M_Boredom proneness**	3.50 (± 1.57)	3.44 (± 1.52)	2.72 (± 1.57)	3.07 (± 1.61)	***F* = 11.15, ***p*** < 0.001** **	*F* = 0.675, *p* = 0.412	*F* = 1.425, *p* = 0.233
M_Concentration tendency	5.06 (± 1.42)	5.13 (± 1.35)	5.00 (± 1.66)	5.31 (± 1.60)	*F* = 0.130, *p* = 0.718	*F* = 1.337, *p* = 0.248	*F* = 0.492, *p* = 0.484
M_ADHD tendency	21.33 (± 9.52)	19.83 (± 8.02)	19.83 (± 8.63)	19.85 (± 9.19)	*F* = 0.571, *p* = 0.450	*F* = 0.567, *p* = 0.452	*F* = 0.593, *p* = 0.442
M_Responsiveness	3.03 (± 0.45)	3.00 (± 0.41)	3.09 (± 0.42)	3.03 (± 0.46)	*F* = 1.566, *p* = 0.212	*F* = 1.617, *p* = 0.204	*F* = 0.006, *p* = 0.938
**M_Control**	3.47 (± 0.34)	3.41 (± 0.35)	3.55 (± 0.33)	3.53 (± 0.35)	***F* = 7.458, ***p*** = 0.007** **	*F* = 1.163, *p* = 0.282	*F* = 0.285, *p* = 0.594

M_: mother’s. C_: child’s. M_BPS: BPS scale score for mother. M_Responsiveness: “Responsiveness” subscale score for mother’s parenting style, M_Control: “Control” subscale score for mother’s parenting style. Values other than the results of F-test represent mean values (± SD). Bold values represent statistically significant or marginally significant effects. **p ≤ 0.01,*p ≤ 0.05.

**Table 2:** Descriptive statistics for online-play and non-online-play groups.

Dependent variable	Group				*F(*χ^2^*)* and *p* values		
	Online-play group		Non-online-play group		Main group effect	Main gender effect	Interaction
	Boys	Girls	Boys	Girls			
Gender (number)	*n* = 65	*n* = 30	*n* = 107	*n* = 139	*χ^2^* = 17.03, ***p*** < 0.001** (Distribution of the numbers of boys and girls)		
**C_Boredom proneness**	4.51 (± 1.72)	4.00 (± 1.60)	3.66 (± 1.79)	3.83 (± 1.76)	***F* = 5.194, ***p*** = 0.023** *	*F* = 0.594, *p* = 0.441	*F* = 2.265, *p* = 0.133
**C_Concentration tendency**	5.49 (± 1.50)	5.07 (± 1.41)	5.77 (± 1.20)	5.63 (± 1.19)	***F* = 6.711, ***p*** = 0.010** **	*F* = 2.968, *p* = 0.086	*F* = 0.812, *p* = 0.368
**C_ADHD tendency**	13.17 (± 11.70)	9.10 (± 7.05)	10.40 (± 10.26)	7.24 (± 7.30)	***F* = 3.857 ***p*** = 0.050** *	***F* = 9.414, ***p*** = 0.002** **	*F* = 0.147, *p* = 0.701
**M_BPS**	102.22 (± 16.64)	104.40 (± 14.64)	98.36 (± 15.48)	97.99 (± 14.65)	***F* = 6.894, ***p*** = 0.009** **	*F* = 0.217, *p* = 0.642	*F* = 0.424, *p* = 0.515
M_Boredom proneness	3.55 (± 1.50)	3.30 (± 1.69)	3.00 (± 1.65)	3.24 (± 1.55)	*F* = 2.266, *p* = 0.133	*F* = 0.001, *p* = 0.982	*F* = 1.517, *p* = 0.219
M_Concentration tendency	5.18 (± 1.33)	5.10 (± 1.23)	4.94 (± 1.61)	5.24 (± 1.52)	*F* = 0.063, *p* = 0.802	*F* = 0.320, *p* = 0.572	*F* = 1.017, *p* = 0.314
M_ADHD tendency	21.69 (± 8.34)	20.77 (± 10.17)	20.20 (± 9.67)	19.64 (± 8.25)	*F* = 1.327, *p* = 0.250	*F* = 0.424, *p* = 0.516	*F* = 0.026, *p* = 0.871
M_Responsiveness	3.04 (± 0.43)	2.90 (± 0.36)	3.06 (± 0.45)	3.02 (± 0.44)	*F* = 1.588, *p* = 0.208	*F* = 2.601, *p* = 0.108	*F* = 0.774, *p* = 0.379
**M_Control**	3.45 (± 0.30)	3.34 (± 0.34)	3.53 (± 0.35)	3.50 (± 0.35)	***F* = 7.709, ***p*** = 0.006** **	*F* = 2.609, *p* = 0.107	*F* = 0.690, *p* = 0.407

Abbreviations and presentation format are similar to those in Table 1. Bold values represent statistically significant or marginally significant effects. **p ≤ 0.01, *p ≤ 0.05.

Significant differences between the online-concentrated and non-online-concentrated groups are shown in Table 1. A significant gender difference was observed only for ADHD tendencies [*F*(1, 337) = 11.16, *p* < 0.001, *η_p_*^2^ = 0.032], with no significant interaction between group and gender for any variable. Significant group differences were found in children’s boredom [*F*(1, 337) = 22.52, *p* < 0.001, *η_p_*^2^ = 0.063], concentration [*F*(1, 337) = 19.10, *p* < 0.001, *η_p_*^2^ = 0.054]), and ADHD tendencies [*F*(1, 337) = 11.60, *p* < 0.001, *η_p_*^2^ = 0.033]. Children in the online-concentrated group showed significantly higher boredom and ADHD tendencies, whereas children in the non-online-concentrated group demonstrated significantly higher concentration tendencies. Mothers of children in the online-concentrated group also reported significantly higher boredom, both on the BPS [*F*(1, 337) = 7.32, *p* = 0.007, *η_p_*^2^ = 0.021] and the single-item rating scale [*F*(1, 337) = 11.15, *p* < 0.001, *η_p_*^2^ = 0.032]. In contrast, mothers of children in the non-online-concentrated group scored significantly higher on the “control” dimension of parenting [*F*(1, 337) = 7.46, *p* = 0.007, *η_p_*^2^ = 0.022].

Table 2 presents the group differences between the online-play and non-online-play groups. A significant gender difference was found only for ADHD tendencies [*F*(1, 337) = 9.41, *p* = 0.002, *η_p_*^2^ = 0.027], with no significant interaction between group and gender for any variable. Children in the online-play group exhibited significantly higher levels of boredom [*F*(1, 337) = 5.19, *p* = 0.023, *η_p_*^2^ = 0.015] and ADHD tendencies [*F*(1, 337) = 3.86, *p* = 0.050, *η_p_*^2^ = 0.011], whereas children in the non-online-play group demonstrated significantly greater concentration tendencies [*F*(1, 337) = 6.71, *p* = 0.010, *η_p_*^2^ = 0.020]. Mothers of children in the online-play group reported significantly higher levels of boredom as measured by the BPS [*F*(1, 337) = 6.89, *p* = 0.009, *η_p_*^2^ = 0.020]. In contrast, mothers of children in the non-online-play children had significantly higher scores on the “control” dimension of parenting [*F*(1, 337) = 7.71, *p* = 0.006, *η_p_*^2^ = 0.022].

### 3.3 Binomial logistic regression analyses

To identify significant predictors of group classification (online- vs. non-online-concentrated and online- vs. non-online-play), binomial logistic regression analyses were conducted. The independent variables included those listed in Tables 1 and 2, and the dependent variable was group classification. Although several variables were significantly correlated, the correlation coefficients were relatively low; therefore, all variables were retained in the analyses. Both models showed good fit and significance (Table 3). Correlation matrices are available in the Supplementary material.

**Table 3:** Odds ratios for the independent variables in binomial logistic regression analyses.

Dependent variable	Independent variable	Odds ratio (OR)	95% CI of OR	Wald	*p*-value
Online-concentrated group or Non-online-concentrated group	**Gender**	0.593	0.368, 0.954	**4.640**	0.031*
	**C_Boredom tendency**	1.159	0.996, 1.349	**3.648**	**0.056** ^**†**^
	**C_Concentration tendency**	0.774	0.622, 0.963	**5.265**	0.022*
	**C_ADHD tendency**	1.041	1.007, 1.077	**5.687**	0.017*
	M_BPS	1.005	0.986, 1.024	0.235	0.628
	**M_Boredom tendency**	1.189	0.999, 1.415	**3.818**	**0.051** ^**†**^
	M_Concentration tendency	1.049	0.891, 1.235	0.324	0.569
	**M_ADHD tendency**	0.970	0.940, 1.001	**3.558**	**0.059** ^**†**^
	M_Responsiveness	1.086	0.599, 1.968	0.074	0.785
	**M_Control**	0.417	0.200, 0.867	**5.483**	0.019*
Omnibus test of model coefficients	χ^2^ = 51.56, ***p*** < 0.001**	Hosmer-Lemeshow test	χ^2^ = 7.770, *p* = 0.456	Nagelkerke R^2^ = 0.188	
Online-play group or Non-online-play group	**Gender**	0.337	0.197, 0.579	**15.581**	< 0.001**
	C_Boredom tendency	1.108	0.938, 1.309	1.467	0.226
	C_Concentration tendency	0.891	0.718, 1.107	1.089	0.297
	C_ADHD tendency	1.015	0.985, 1.047	0.961	0.327
	M_BPS	1.016	0.995, 1.038	2.277	0.131
	M_Boredom tendency	1.031	0.855, 1.243	0.104	0.747
	M_Concentration tendency	1.142	0.945, 1.381	1.889	0.169
	M_ADHD tendency	0.991	0.959, 1.024	0.299	0.584
	M_Responsiveness	1.062	0.559, 2.019	0.034	0.854
	**M_Control**	0.398	0.184, 0.862	**5.455**	0.020*
Omnibus test of model coefficients	χ^2^ = 38.05, ***p*** < 0.001**	Hosmer-Lemeshow test	χ^2^ = 11.86, *p* = 0.158	Nagelkerke R^2^ = 0.152	

Abbreviations and presentation format are similar to those in Tables 1 and 2. Bold values represent statistically significant or marginally significant effects. **p ≤ 0.01, *p ≤ 0.05, ^†^*p* ≤ 0.1.

As shown in Table 3, classification into the non-online-concentrated group was significantly associated with being female (odds ratio [OR] = 0.593, 95% confidence interval [CI] [0.368–0.954], *p* = 0.031), child’s concentration tendencies (OR = 0.774, 95% CI [0.622–0.963], *p* = 0.022), and maternal control (OR = 0.417, 95% CI [0.200–0.867], *p* = 0.019). Conversely, higher ADHD tendencies significantly increased the likelihood of classification into the online-concentrated group (OR = 1.041, 95% CI [1.007–1.077], *p* = 0.017). Marginal significant predictors for online-concentrated group classification included child boredom (OR = 1.159, 95% CI [0.996–1.349], *p* = 0.056), maternal boredom (OR = 1.189, 95% CI [0.999–1.415], *p* = 0.051), and maternal ADHD tendencies (OR = 0.970, 95% CI [0.940–1.001], *p* = 0.059).

For classification into the non-online-play group, only being female (OR = 0.337, 95% CI [0.197–0.579], *p* < 0.001) and higher maternal control (OR = 0.398, 95% CI [0.184–0.862], *p* = 0.020) were significant predictors.

## 4 Discussion

In both comparisons of online versus non-online groups, children in the online groups exhibited higher levels of boredom and ADHD tendencies, as well as greater maternal boredom tendencies. In contrast, children in the non-online groups demonstrated higher concentration tendencies, while their mothers reported higher levels of parental control.

Although the significant predictors identified in the regression analysis for classifying children into online- and non-online-concentrated groups closely mirrored the findings from the descriptive analyses, only two significant predictors emerged in the analysis of the play-based groups. This discrepancy may be due to the uneven distribution of participants in the online (*n* = 95) and non-online play groups (*n* = 246), suggesting that, up to the age of 10 years, parental control may play a substantial role in limiting children’s engagement with digital and online play. Conversely, a relatively large proportion of children (*n* = 191) were classified into the online-concentrated group, implying that an orientation toward online content may develop in early childhood, even when daily screen use is restricted.

ADHD and boredom tendencies, both previously associated with IA, were also linked to an online orientation and preference in relatively young children. Notably, maternal boredom tendencies were correlated with children’s online orientation, while greater maternal control was linked to a non-online orientation. Mothers who are more prone to boredom may have difficulty managing it through offline activities and may themselves exhibit a preference for online engagement. These maternal patterns may, in turn, contribute to the development of the child’s orientation toward online content. This interpretation remains speculative and requires further empirical investigation.

Previous studies have suggested that PS influences IA in adolescence; however, the most effective parenting strategies for preventing IA remain unclear. Nonetheless, these findings highlight the importance of parental involvement in shaping children’s online behaviors. Our results suggest that parents should regulate their children’s online activities based on individual characteristics—such as boredom and ADHD tendencies—while also considering their own predispositions, including boredom tendencies, which may influence their children’s orientation toward online engagement.

However, this study has several limitations, including its reliance solely on mothers’ reports, a limited number of measured variables, and the absence of an examination of IA’s impact on other domains. Future research should collect a broader range of data related to IA from both parents and children and investigate the bidirectional relationship between IA and other variables.

Despite the exploratory nature of this study, it offers valuable insights into online orientation among young children. Given the increasing prevalence of online activities, including the use of AI, it is evident that children—particularly those exhibiting high levels of ADHD and boredom tendencies—are naturally drawn to online activities. From a family perspective, it is important to examine how parental characteristics (e.g., boredom and ADHD tendencies) and parental attitudes toward children influence their vulnerability to IA. Further, understanding how these parental variables interact with children’s individual traits could inform prevention strategies tailored to mitigate IA risk. From an educational standpoint, future research should focus on strategies that leverage online platforms to support children’s learning and well-being, while simultaneously minimizing the risk of IA. Given the possibility that independent concentration ability may serve as a protective factor against IA, it appears essential to foster children’s capacity to autonomously select and reject online activities based on their potential to enhance learning and well-being.

## Data Availability

The raw data supporting the conclusions of this article will be made available by the authors, without undue reservation.
